# A pan-cancer analysis revealing the role of TIGIT in tumor microenvironment

**DOI:** 10.1038/s41598-021-01933-9

**Published:** 2021-11-18

**Authors:** Jie Wen, Xueyi Mao, Quan Cheng, Zhixiong Liu, Fangkun Liu

**Affiliations:** 1 Department of Neurosurgery, Xiangya Hospital, Central South University, Changsha, 41008 China; 2 Department of Clinical Pharmacology, Xiangya Hospital, Central South University, Changsha, China; 3 National Clinical Research Center for Geriatric Disorders, Xiangya Hospital, Central South University, Changsha, China

**Keywords:** Immunotherapy, Tumour immunology, Cancer, Cancer microenvironment, Tumour immunology, Prognostic markers, Biomarkers, Drug development

## Abstract

T cell immunoreceptor with immunoglobulin and ITIM domain (TIGIT), an immune checkpoint, plays a pivotal role in immune suppression. However its role in tumor immunity and correlation with the genetic and epigenetic alterations remains unknown. Here, we comprehensively analyzed the expression patterns of the TIGIT and its value of prognostic prediction among 33 types of cancers based on the data collected from The Cancer Genome Atlas (TCGA) and the Genotype-Tissue Expression projects (GTEx). Furthermore, the correlations of TIGIT with pathological stages, tumor-infiltrating immune cells (TIICs), signatures of T cells subtypes, immune checkpoint genes, the degree of Estimation of STromal and Immune cells in MAlignant Tumor tissues using the Expression data (ESTIMATE), tumor mutation burden (TMB), microsatellite instability (MSI), mismatch repair (MMR) genes, and DNA methyltransferases (DNMTs) were also explored. Gene functional enrichment was conducted by Gene Set Enrichment Analysis (GSEA). Our results showed that the expression of TIGIT was upregulated in most of the cancer types. Cox regression model showed that high expression of TIGIT in tumor samples correlates with poor prognosis in KIRC, KIRP, LGG, UVM, and with favorable prognosis in BRCA, CECS, HNSC, SKCM. TIGIT expression positively correlated with advanced stages, TIICs, the signatures of effector T cells, exhausted T cells, effector Tregs and the degree of ESTIMATE in KIRC, KIRP and UVM. TIGIT expression also positively correlated with CTLA4, PDCD1 (PD-1), CD274 (PD-L1), ICOS in most of the cancer types. Furthermore, the expression of TIGIT was correlated with TMB, MSI, MMR genes and DNMTs in different types of cancers. GSEA analysis showed that the expression of TIGIT was related to cytokine-cytokine receptor interaction, allograft rejection, oxidative phosphorylation. These findings suggested that TIGIT could serve as a potential biomarker for prognosis and a novel target for immunotherapies in cancers.

## Introduction

T cell immunoreceptor with immunoglobulin and ITIM domain (TIGIT, also called WUCAM, Vstm3, VSIG9), an immune inhibitory receptor (IR) and immune checkpoint expressed on NK cells and T cells including CD4^+^ T cells, CD8^+^ cells and T_regs_, plays critical roles in limiting adaptive and innate immunity against tumors^1–3^. Upregulation of TIGIT was observed in a variety of cancers, such as lung cancers^4^, kidney cancers^5^, liver cancers^6^. Several mechanisms of the TIGIT inhibition of T cells in the tumor microenvironment (TME) have been revealed^7^. Currently, TIGIT has been viewed as a promising biomarker to predict the prognosis and a potential target to develop novel immunotherapies^8^. However, a specific function of TIGIT in pan-cancers remains largely unknown.

The initiation and development of the cancers are largely dependent on immune dysfunction^9^.TME consists of a variety of cells including immune cells, stromal cells, etc. The tumor and immune cells interact with each other dynamically in TME, which determines the characteristics and heterogenicity of the cancers^10,11^. Under chronic exposure to tumor antigens, T cells become dysfunctional/exhausted and upregulate several IRs including programmed cell death receptor 1 (PD-1) and TIGIT^12,13^. Immunotherapies such as Immune checkpoint blockades (ICBs) have achieved great progress and show tremendous potentiality, especially for those patients with resistance to chemoradiotherapy^14,15^. However, the clinical options of the immunotherapies are still lacking^16^. Thus it is of great significance and urgency to explore and validate more effective immune-related targets.

In this research, taking advantages of TCGA and GTEx datasets, we conducted a comprehensive analysis at pan-cancer level to illustrate the TIGIT expression profiles, prognostic values and its correlation with immune infiltration level, tumor mutation burden (TMB), microsatellite instability (MSI), mismatch repair (MMR) genes, and DNA methyltransferases (DNMTs).

## Methods

### Data source and processing

The TIGIT expression data of 33 types of cancers and corresponding clinical information were acquired from The Cancer Genome Atlas through the UCSC cancer genome browser (https://tcga.xenahubs.net, accessed April 2020)^17^. To compare with the TIGIT expression level in normal tissues, we extracted RNA sequences in normal tissues from Genotype-tissue expression (GTEx; http://commonfund.nih.gov/GTEx/).

Data of 33 types of cancer were introduced into the final analysis, including Adrenocortical Carcinoma (ACC), Bladder Urothelial Carcinoma (BLCA), Breast invasive carcinoma (BRCA), Cervical squamous cell carcinoma and endocervical adenocarcinoma (CESC), Cholangiocarcinoma (CHOL), Colon adenocarcinoma (COAD), Lymphoid Neoplasm Diffuse Large B-cell Lymphoma (DLBC), Esophageal carcinoma (ESCA), Glioblastoma multiforme (GBM), Head and Neck squamous cell carcinoma (HNSC), Kidney Chromophobe (KICH), Kidney renal clear cell carcinoma (KIRC), Kidney renal papillary cell carcinoma (KIRP), Acute Myeloid Leukemia (LAML), Brain Lower Grade Glioma (LGG), Liver hepatocellular carcinoma (LIHC), Lung adenocarcinoma (LUAD), Lung squamous cell carcinoma (LUSC), Mesothelioma (MESO), Ovarian serous cystadenocarcinoma (OV), Pancreatic adenocarcinoma (PAAD), Pheochromocytoma and Paraganglioma (PCPG), Prostate adenocarcinoma (PRAD), Rectum adenocarcinoma (READ), Sarcoma (SARC), Skin Cutaneous Melanoma (SKCM), Stomach adenocarcinoma (STAD), Testicular Germ Cell Tumors (TGCT), Thyroid carcinoma (THCA), Thymoma (THYM), Uterine Corpus Endometrial Carcinoma (UCEC), Uterine Carcinosarcoma (UCS), Uveal Melanoma (UVM).

### Gene expression and survival analysis

The TIGIT expression data of 33 cancer types from TCGA and normal samples from GTEx were extracted and formed an expression matrix. Using univariate cox model to evaluate the correlation between TIGIT expression and patient survival for the 33 cancer types. Based on the median TIGIT expression levels, we stratified patients into the high and low group. The Kaplan–Meier (KM) analysis by log rank test was applied to compared patient prognosis from these 2 groups. A p < 0.05 was considered as statistical significance.

GEPIA2 (Gene Expression Profiling Interactive Analysis 2, http://gepia2.cancer-pku.cn/#index) was a powerful resource for analysis of gene expression based on the data from TCGA and GTEx database^18^. Here we assessed the correlation between TIGIT expression and pathological stages in cancers by utilizing GEPIA2.

### Relationship between TIGIT expression and immunity

We explored the abundance of tumor-infiltrated immune cells (TIICs) among 33 types of cancers through Tumor Immune Estimation Resource (TIMER, https://cistrome.shinyapps.io/timer/)^19^ and Cell-type identification by Estimating Relative Subsets of RNA Transcripts (CIBERSORT)^20^ respectively. The correlation between the TIGIT expression level and the abundance of TIICs including CD4+ T cells, CD8+ T cells, B cells, neutrophils, macrophages and dendritic cells. GEPIA2 was utilized to analyze the correlation between TIGIT expression and signatures of T cells subtypes. We chose log2 transformed expression data as parameter selection for plotting.

We also utilized the Estimation of STromal and Immune cells in MAlignant Tumor tissues using Expression data (ESTIMATE) to generate 3 scores including stromal score, immune score, and ESTIMATE score, which represented the immunocyte infiltration level, stromal cells, tumor purity respectively in tumor tissues^21^. We further analyzed the correlation between TIGIT expression and these 3 scores.

In addition, to explore the potential mechanism of immune inhibition of TIGIT signaling, the correlations of TIGIT expression with other checkpoint markers were compared across diverse cancer types with preference to previous researches^22–24^, with the generation of estimated statistical significance and Spearman’s correlation coefficient.

Through the extraction of somatic mutation profiles of all patients from TCGA, we calculated the TMB scores, MSI scores and analyzed their correlation with TIGIT expression. We also conducted correlation analysis between TIGIT expression and MMR genes, DNMTs, respectively. We drew the figures to visualization of the results on Sangerbox online platform (http://sangerbox.com/).

### Gene set enrichment analysis

To explore the biological signaling pathway of TIGIT, gene set enrichment analysis (GSEA) was performed by KEGG and HALLMARK analyses^25–27^. We acquired the permission to use the KEGG software from the Kanehisa laboratories. Significant enrichment results were demonstrated using normalized enrichment scores (NES), gene ratio and p value^28^. A p < 0.05 and FDR ≤ 0.25 were considered as statistical significance.

### Statistical analysis

Gene expression profiles acquired from TCGA and GTEx were analysed by Students’ t-test. Spearman’s correlation analysis was applied to evaluated the correlation between TIGIT expression and the abundance of TIICs and scores of immune cells. All analyses were performed with the R package (ggplot2, circlize, clusterProfiler, DOSE and enrichplot) (http://www.r-project.org/) to visualize the results. A p < 0.05 indicated statistical significance.

## Results

### Pan-cancer expression landscape of TIGIT

**Figure 1:**
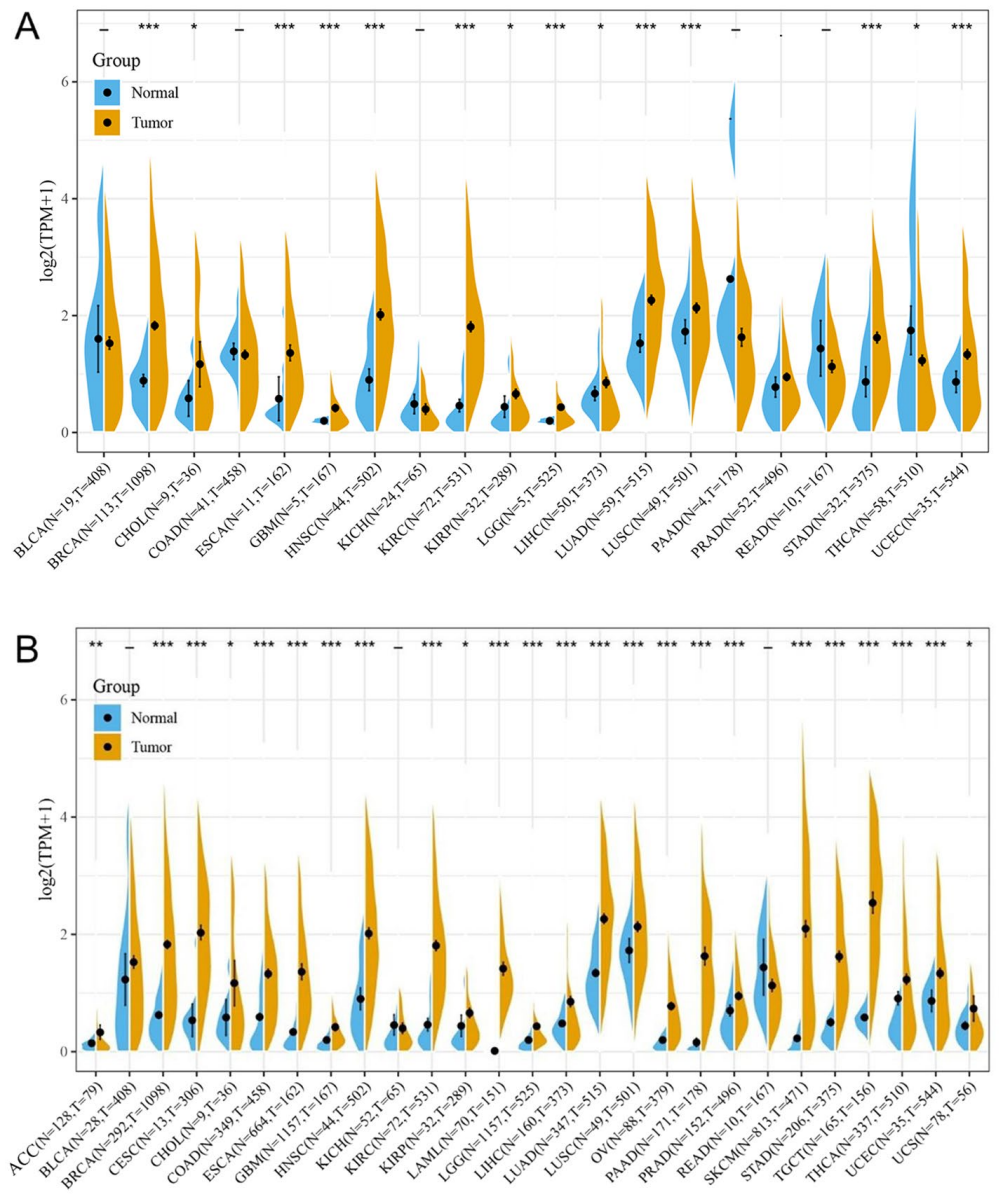
TIGIT expression levels in different types of human cancers. The expression level of TIGIT between tumor and normal tissues were compared in twenty cancer types based on the TCGA database (**A**) and twenty-seven cancer types based on the integrated database from TCGA and GTEx datasets (**B**). *p < 0.05, **p < 0.01, and ***p < 0.001.

Comparison of expression of TIGIT between normal and tumor samples across TCGA cancer types and the combined datasets based on integrated database of GTEx and TCGA datasets were conducted and showed in Fig. 1. Consistent upregulated expression of TIGIT were seen in BRCA, CHOL, ESCA, GBM, HNSC, KIRC, KIRP, LGG, LIHC, LUAD, LUSC, STAD, USEC compared with normal tissues based on both comparisons as shown in Fig. 1A,B. The TIGIT expression was downregulated in THCA based on TCGA datasets. On the contrary, the integrated database showed that TIGIT expression was significantly higher in THCA than in normal tissues. Besides THCA, patients with ACC, CESC, COAD, LAML, OV, PAAD, PRAD, TGCT also exhibited significantly higher expression of TIGIT in integrated database.

The expression level of TIGIT in tumor samples of BRCA, CHOL, ESCA, GBM, HNSC, KIRP, KIRC, LGG, LIHC, LUAD, LUSC, STAD were significantly higher than in normal tissues based on both comparisons as shown in Fig. 1A,B, and the expression level of TIGIT were also significantly increased in ACC, BLCA, CESC, CHOL, COAD, ESCA, GBM, HNSC, KIRC, LAML, LGG, LUSC, OV, PAAD, PRAD, SKCM, STAD, TGCT, THCA, UCS compared with normal tissues based on the integrated database.

### Prognostic value of TIGIT in cancers

**Figure 2:**
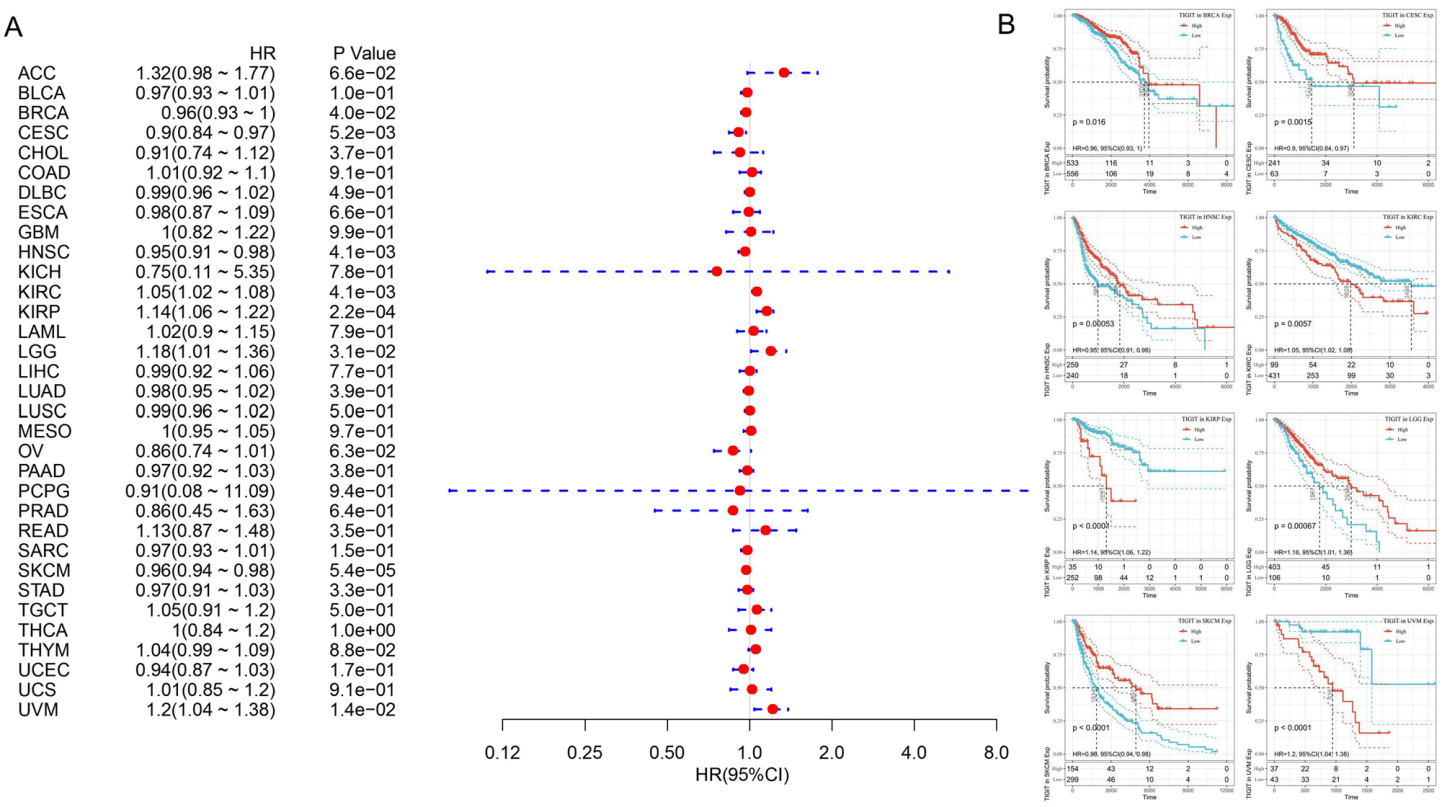
Selected Kaplan–Meier plots and forest plot comparing the high and low expression of TIGIT on overall survival (OS) across different cancers (**A**) Forest plot exhibiting the influence of high expression of TIGIT on OS across thirty three cancer types using Cox regression model. (**B**) Kaplan–Meier survival curves comparison of high and low expression of TIGIT for the OS analysis for BRCA, CECS, HNSC, KIRC, KIRP, LGG, SKCM and UVM.

**Figure 3:**
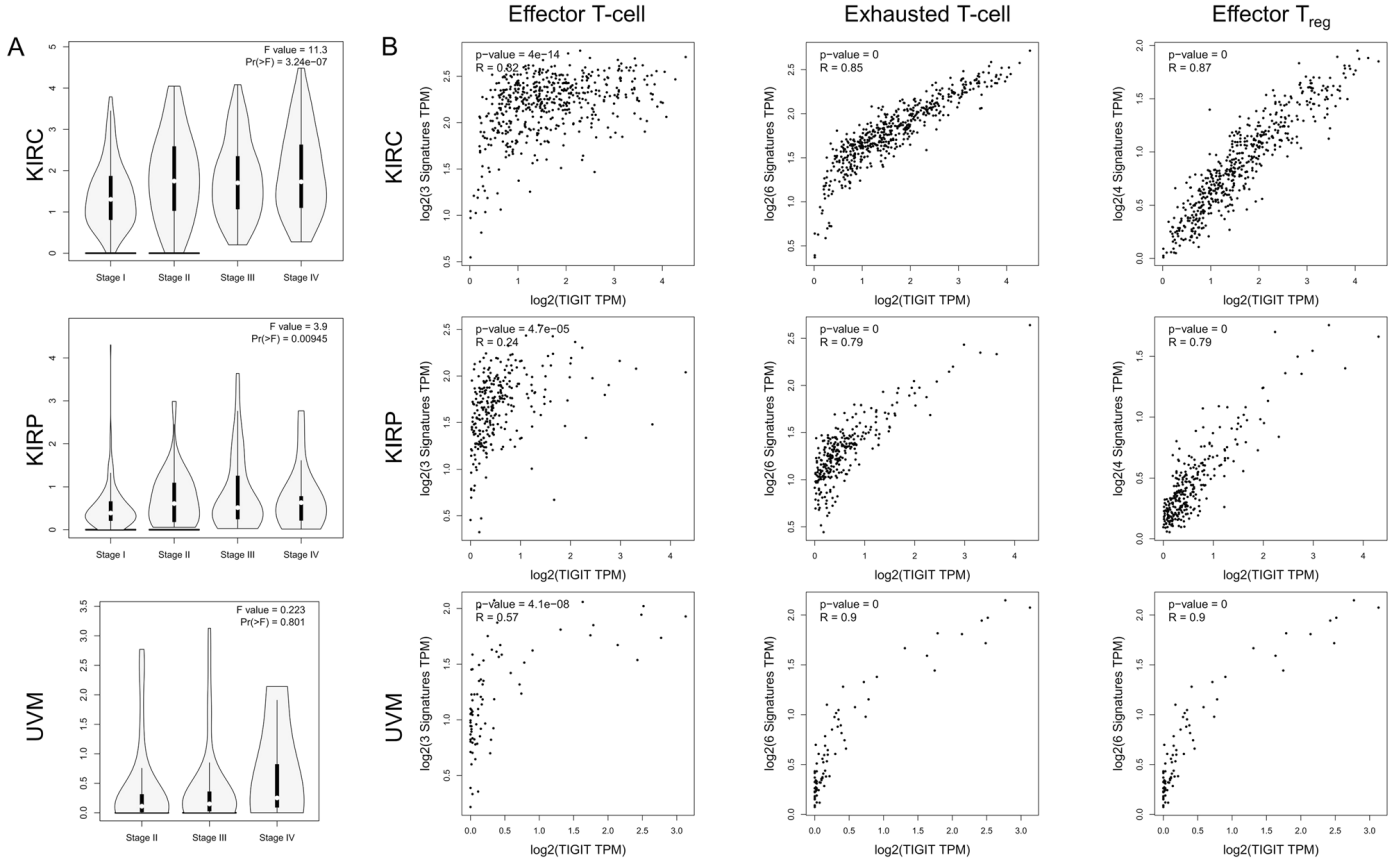
The correlations of TIGIT with pathological stages (**A**) and the signatures of effector T cells, exhausted T cells and effector T_regs_ (**B**) in KIRC, KIRP, UVM by GEPIA2 analysis.

Figure 2 summarized the results of overall survival (OS) analyses of TIGIT expression across the 33 cancer types. Cox regression model showed that high expression of TIGIT in tumor samples correlates with poor prognosis in KIRC (HR, 1.05, 95% CI 1.02–1.08), KIRP (HR, 1.14, 95% CI 1.06–1.22), LGG (HR, 1.18, 95% CI 1.01–1.36), UVM (HR, 1.2, 95% CI 1.04–1.38), and with favorable prognosis in BRCA (HR, 0.96, 95% CI 0.93–1), CECS (HR, 0.9, 95% CI 0.84–0.97), HNSC (HR, 0.95, 95% CI 0.91–0.98), SKCM (HR, 0.96, 95% CI 0.94–0.98) (Fig. 2A). Univariate analysis confirmed the prognostic impact of TIGIT in KIRC (p = 0.0057), KIRP (p < 0.0001) and UVM (p < 0.0001) with the same trend (Fig. 2B). In addition, based on the GEPIA2 dataset, we verified that TIGIT expression had a forceful positive correlation with advanced cancer stages in KIRC, KIRP and SKCM (p < 0.01, Fig. 3A and Supplementary File 1). More information was available in the Supplementary File 1.

### Correlation between TIGIT and immune infiltration level

**Figure 4:**
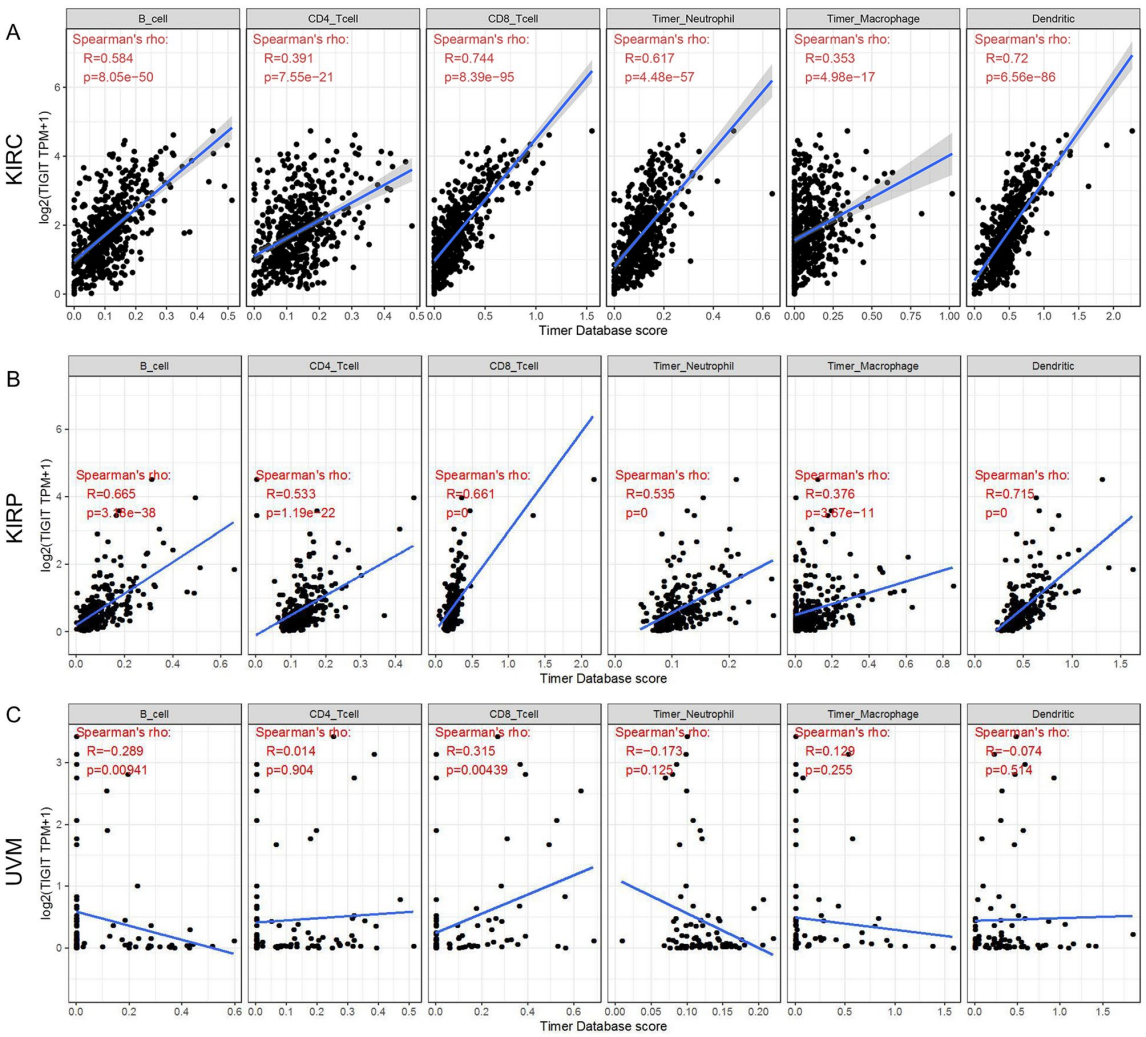
Correlation of TIGIT expression with immune infiltration level in KIRC, KIRP and UVM (**A**–**C**).

Considering several studies have revealed the regulatory function of TIGIT in TME, we analyzed its effect on the abundance of immune infiltration levels in tumors that harbor prognostic value. TIMER showed that TIGIT positively correlated with the abundance of CD8+ T cell in KIRC, KIRP, UVM and also positively correlated with the abundance of B cell, CD4+ T cell, Neutrophil, Macrophage and Dendritic cell in KIRC, KIRP, while TIGIT negatively correlated with the abundance of B cell in UVM (Fig. 4). These results suggested the association between TIGIT and immune cells infiltration, which might influence the progression of the tumors and patients’ prognoses.

As activated and exhausted T cells would upregulate the expression of TIGIT, we assessed the correlation between TIGIT expression and the signatures of effector T cells, exhausted T cells and effector T_regs_. Similar to TIMER data analysis, we also found that there was a significant positive correlation of TIGIT with the signatures of effector T cells (CX3CR1, FGFBP2, FCGR3A), exhausted T cells (HAVCR2, TIGIT, LAG3, PDCD1, CXCL13, LAYN) and effector T_regs_ (FOXP3, CTLA4, CCR8, TNFRSF9) in KIRC, KIRP, UVM (Fig. 3B), LGG, UVM, BRCA, CECS, HNSC, SKCM (Supplementary File 2).

**Figure 5:**
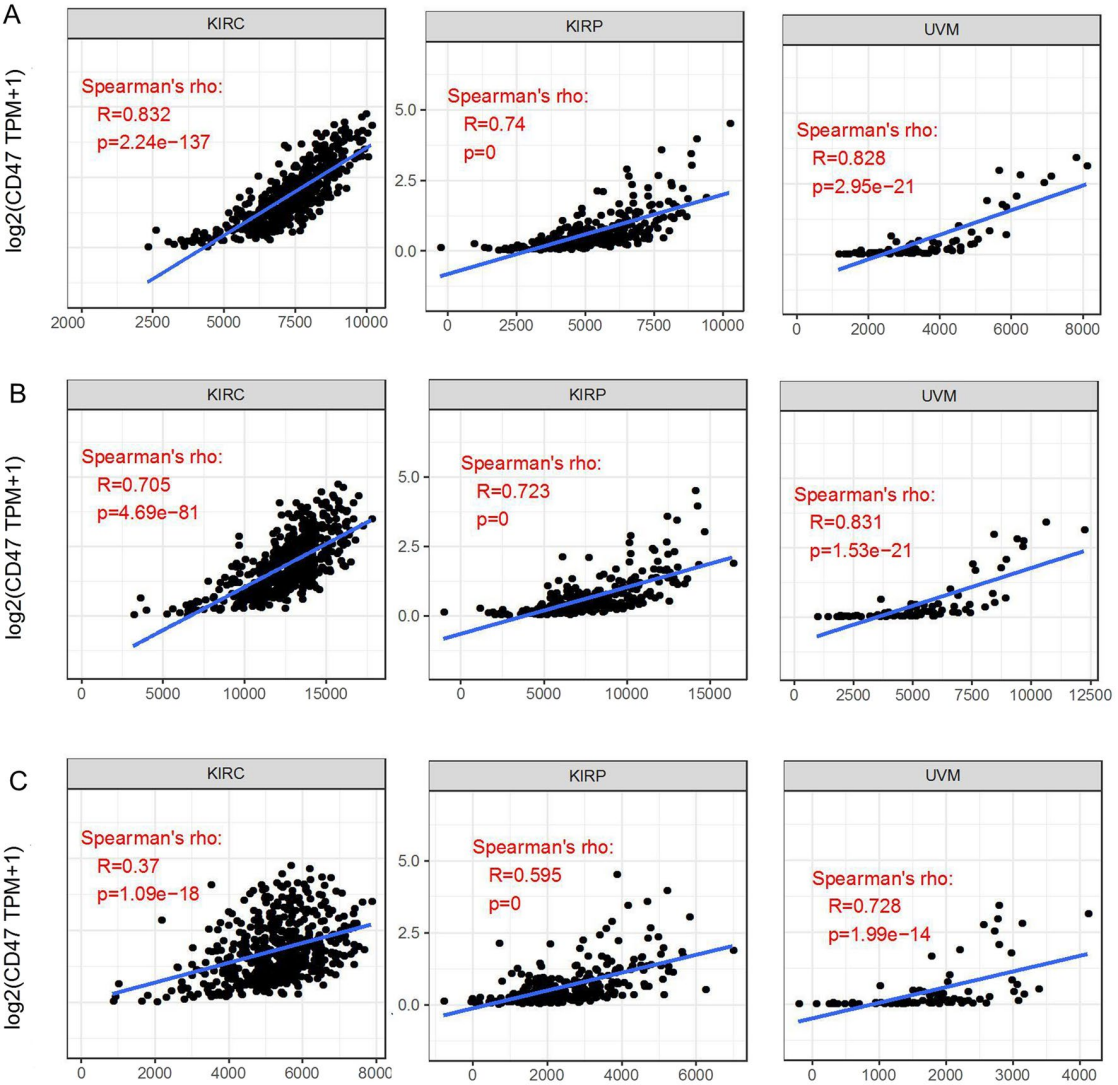
Correlation of TIGIT expression with Immune Score, Estimate Score and Stromal Score in KIRC, KIRP and UVM (**A**–**C**).

We calculated the immune, stromal and estimate scores respectively through ESTIMATE method. Later we evaluated the correlation between TIGIT expression and immune/stromal/estimate scores in three cancer types. As shown in Fig. 5, TIGIT expression was significant correlated with the stromal, immune and estimate scores in all these cancers (all value of p < 0.05). These results indicated that the content of immune or stromal cells elevated and the purity of tumors reduced along with the escalation of the TIGIT expression.

**Figure 6:**
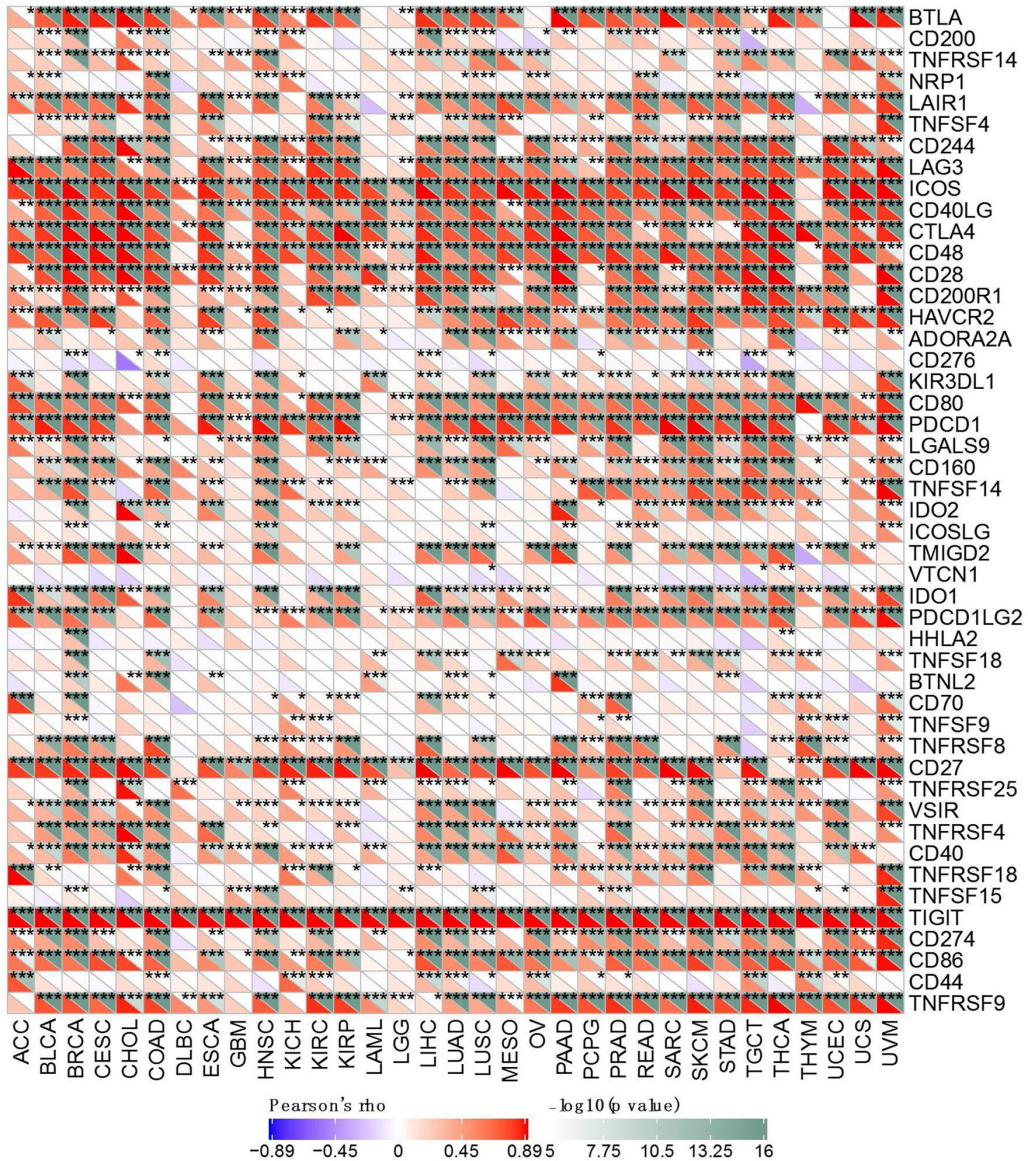
Correlation of TIGIT expression with expression of immune checkpoint genes across 33 cancer types. *p < 0.05, **p < 0.01, and ***p < 0.001.

To further investigate the underlying mechanism of immune inhibition of TIGIT signaling, we analyzed the relationship of TIGIT expression with multiple immune checkpoint markers across 33 cancer types (Fig. 6). Generally, our results showed that TIGIT expression was significantly correlated with many immune checkpoints in diverse immunocytes and distinct T cells, such as the positive correlation of TIGIT with CTLA4, PDCD1 (PD-1), CD274 (PD-L1), ICOS in most of the cancer types, implying a comprehensive co-expressing landscape.

### Correlation analysis on TMB, MSI, MMR and DNMT

**Figure 7:**
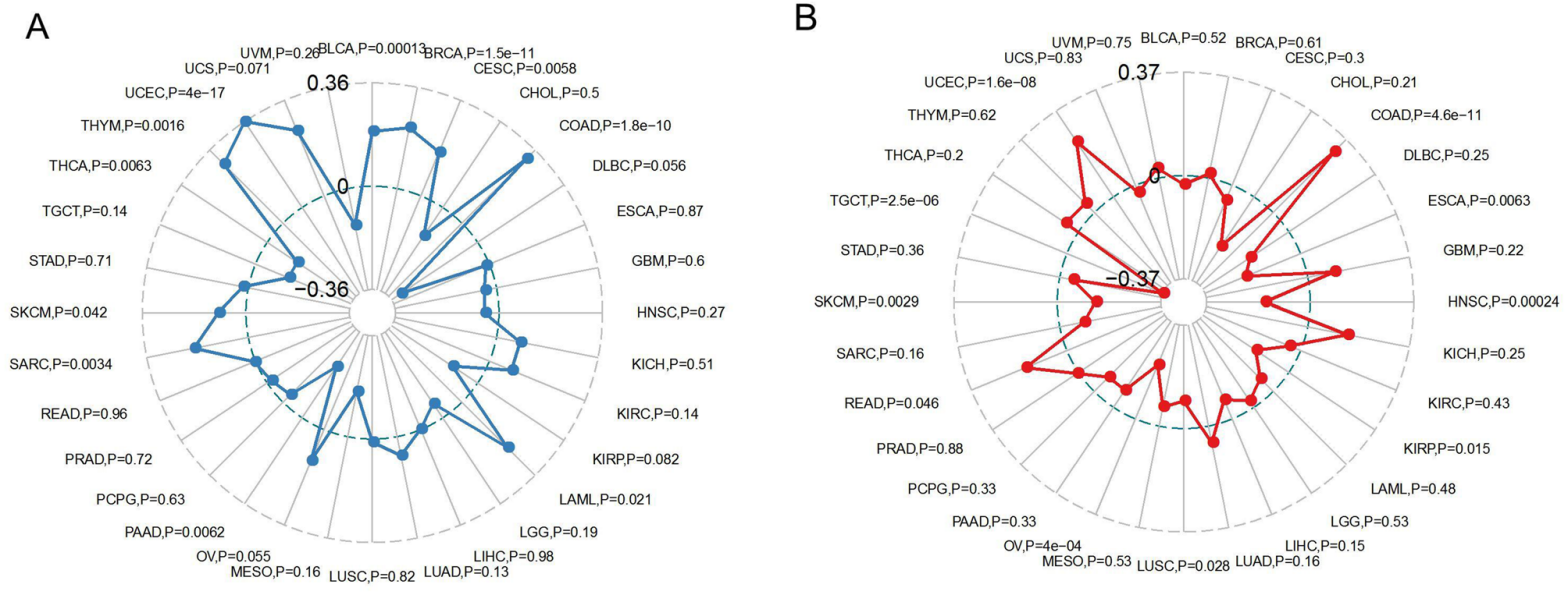
Radar map displaying the correlations between TIGIT expression and Tumor mutation burden (**A**), as well as the correlations between TIGIT expression and microsatellite instability, (**B**) across 33 cancer types.

Moreover, we evaluated the association of TMB/MSI with TIGIT expression (Fig. 7). We found that TIGIT expression was positively correlated with the TMB in BRCA (p < 0.0001), CESC (p = 0.0058), COAD (p < 0.0001), LAML (p = 0.021), SARC (p = 0.0034), SKCM (p = 0.042), THYM (p = 0.0016), UCEC (p < 0.0001), USC (p = 0.071) while negatively correlated with the TMB in BLCA (p = 0.00013), PAAD (p = 0.0062), THCA (p = 0.0063), as shown in Fig. 7A. Moreover, TIGIT expression was found to be positively correlated to the MSI in COAD (p < 0.0001), READ (p = 0.046), UCEC (p < 0.0001) while negatively correlated to the MSI in ESCA (p = 0.0063), HNSC (p = 0.00024), KIRP (p = 0.015), LUSC (p = 0.028), OV (p < 0.0001), SKCM (p = 0.0029), TGCT (p < 0.0001), as presented in Fig. 7B.

**Figure 8:**
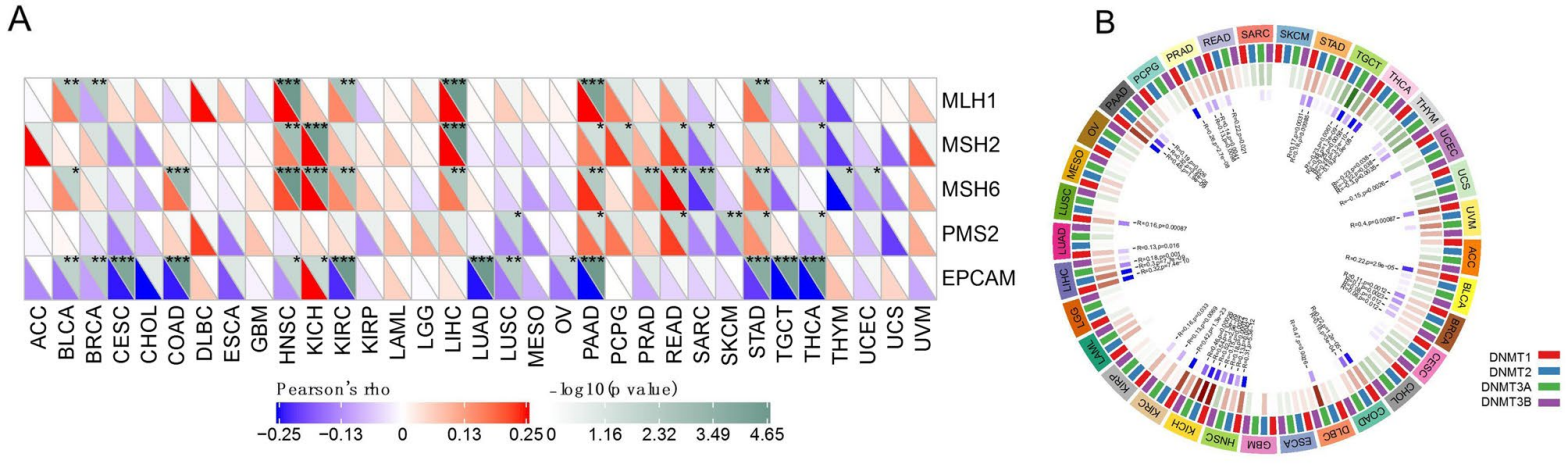
The correlations between TIGIT expression and five mismatch repair genes (**A**), as well as the correlations between TIGIT expression and DNA methyltransferase (**B**) across 33 cancer types. *p < 0.05, **p < 0.01, and ***p < 0.001.

Furthermore, we analyzed the correlation of TIGIT with the expression of MMR genes (MLH1, MSH2, MSH6, PMS2) and EPCAM as the upstream gene of MSH2. In 11 of the 33 cancer types TIGIT was positively correlated with the expression of at least one MMR genes. And in 16 types of cancers TIGIT was negatively correlated with the expression of MMR genes (Fig. 8A). Besides, we also performed a correlation analysis between TIGIT expression and DNMTs expression (DNMT1, DNMT2, DNMT3A, and DNMT3B). As shown in Fig. 8B, TIGIT was positively correlated with at least one DNMTs expression in 17 types cancers, TIGIT was negatively correlated with DNMTs expression in 5 cancer types.

### Functional analysis by GSEA

**Figure 9:**
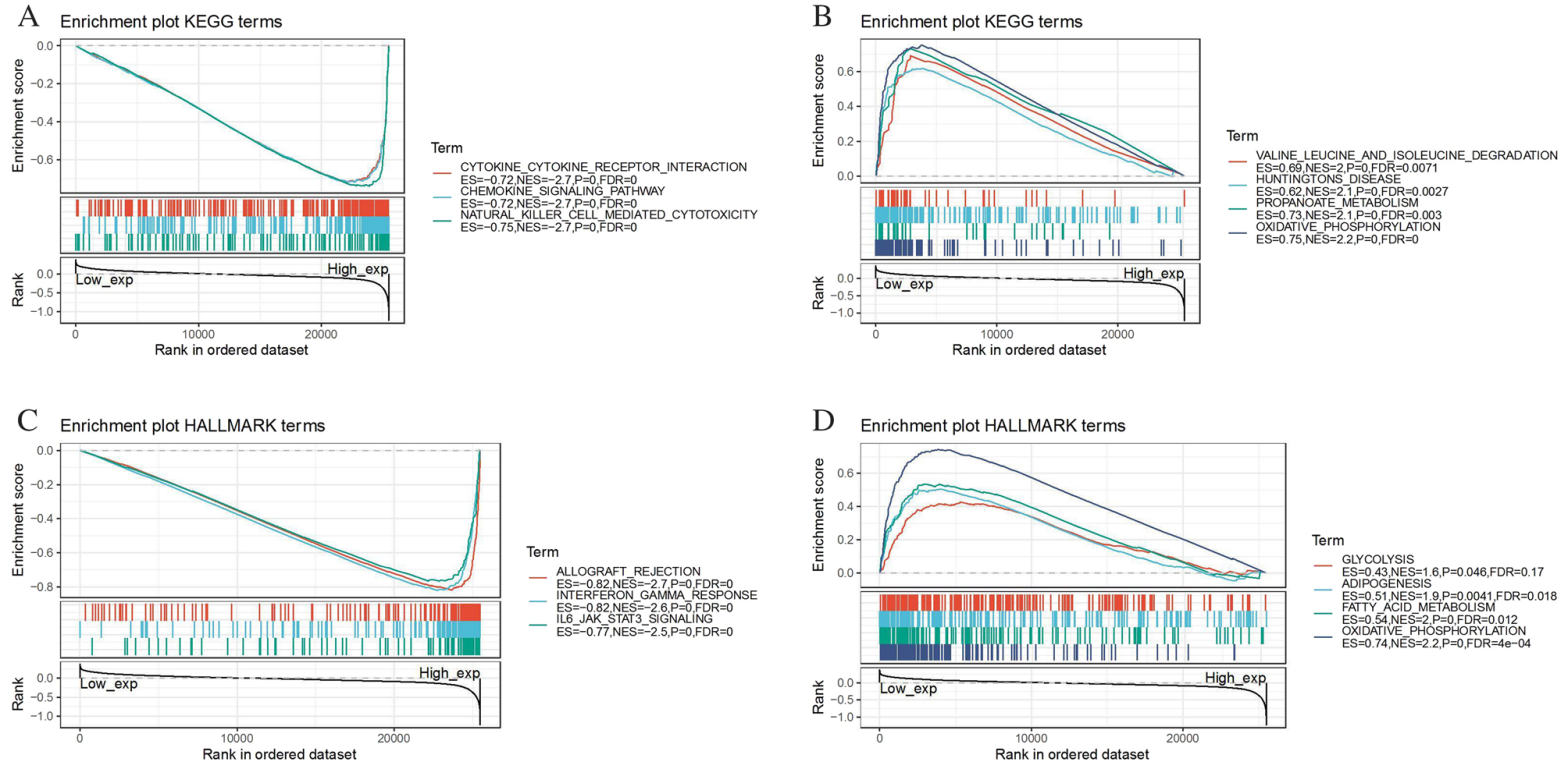
Functional Enrichment of KEGG and HALLMARK terms on TIGIT through GSEA. The top three negative and positive enriched KEGG terms were displayed in (**A**) and (**B**) respectively. The top three negative and positive enriched HALLMARK terms were displayed in (**C**) and (**D**) respectively.

GSEA was performed to explore the biological role of the TIGIT. Generally, the top three negatively enriched KEGG terms in high TIGIT subgroup were cytokine-cytokine receptor interaction, chemokine signaling pathway and natural killer cell mediated cytotoxicity (Fig. 9A) and the top three negatively enriched HALLMARK terms included allograft rejection, interferon-gamma response and IL6-JAK-STAT3 signaling (Fig. 9C). The top positively enriched terms were oxidative phosphorylation and propanoate metabolism (Fig. 9B,D). These results suggested the possible signaling pathway and mechanism of TIGIT function on immune and metabolic function.

## Discussion

The present work illustrated a comprehensive workflow for pan-cancer analysis and thoroughly investigated the role of TIGIT in cancers. The results showed the prognostic impact of TIGIT across the different cancer types. TIGIT expression mediated infiltrated immune cells and positively correlated with the expression of LAG3, CTLA4, PDCD1 (PD-1), CD274 (PD-L1), PDCD1LG2 (PD-L2) in most of the cancer types. TIGIT expression was also correlated with TMB/MSI/DNMTs/MMR genes in multiple cancers. GSEA results demonstrated the high TIGIT patient group negatively enriched terms including cytokine-cytokine receptor interaction, chemokine signaling pathway, natural killer cell mediated cytotoxicity, allograft rejection, interferon gamma response and IL6-JAK-STAT3 signaling.

Our study showed the great prognostic values of TIGIT across different cancer types. Upregulated TIGIT expression has been reported in KIRC^29^, LGG^30^ and correlated to poor prognosis, which was consistent with our results. Our results also revealed the correlation of TIGIT with pathological stages in KIRC, KIRP, and UVM these 3 cancer types with prognostic values. Previous studies and our results both indicated that TIGIT could serve as a potential prognostic biomarker in multiple types of cancers^4,6,31^.

To our knowledge, TIGIT expression would be upregulated along with the activation of T cells and NK cells^3^. In addition, under the chronic antigen stimulation, T cells become dysfunctional/exhausted and would also upregulate the expression of some IRs including TIGIT and PD-1^12,13,32–34^, consistent with our results that TIGIT expression was positively correlated to the effector T cells, exhausted T cells and T_regs_. Several studies have revealed the wide suppressive impact of TIGIT on a wide range of immune cells and immune function^35,36^, generally through (1) binding CD155 and triggering direct inhibitory signals on T/NK cells^3,37^, (2) binding CD155 on APC to produce more anti-inflammatory cytokines^3^, (3) binding CD155 competing with CD226^2^ and disrupting CD226 homodimerization to impede CD226-mediated T cell activation^33^, (4) stabilizing and enhancing the immunosuppressive functions of Tregs^36,38^, (5) binding Fap2 from the gut bacteria Fusobacterium nucleatum and triggering inhibitory signals^39^. The tumor tissues with the upregulated expression of TIGIT also exhibited aberrant immune characteristics. For example, in colorectal tumor tissues, TIGIT+ CD8+ T cells exhibited significantly higher infiltration and an exhausted phenotype with lower expression of proinflammtory cytokines and higher expression of inhibitory receptors such as PD-1, LAG-3, and TIM-3 on the surface^40^. Interestingly, according to our GSEA analysis, TIGIT was also shown the capability for driving the negative regulation on immune-related function and pathway, such as cytokine-cytokine receptor interaction, chemokine signaling pathway and natural killer cell mediated cytotoxicity, interferon-gamma response and IL6-JAK-STAT3 signaling. Our results revealed the positive correlation between TIGIT and TIICs in 3 cancer types with poor prognosis. There are several studies reported that the status of high TIICs may lead to poor prognosis^41,42^, which could be explained that some of the infiltrated immune cells, such as macrophages, would promote or result in tumor initiation, development and metastasis especially in the immunosuppressive microenvironment^41,43^, confirming our findings that TIGIT overexpression is related to the poor prognosis in certain cancers. While TIGIT was negatively correlated with B cells in UVM, contrast to the results in KIRC and KIRP. There was a study reported that high-infiltrated B cells are related to the better prognosis in UVM^41^, which concorded with our results. The discrepancy of infiltrated B cells may result from the different subtypes and the various functions of B cells, which leads to the different prognosis in different cancer types^44,45^. More detailed researches could be further carried on, such as single-cell sequencing. Different results in the same analysis of correlations depending on the cancer types^46^ may attribute to inter-tumor heterogeneity, exhibiting different TME, tumor immunogenicity, TMB and microsatellite states across different caner types^47–49^, which is also the potential mechanism of the discrepancy in response to the ICBs. Given all the information above, it is likely that the immunosuppressive effect of TIGIT leads to the tumor cells survival and escape, influencing the initiation and development of the cancers and the patients’ prognosis.

To further investigate the underlying mechanism of the relationship between TIGIT and tumors, we conducted analyses on the correlation between TIGIT and TMB, MSI, MMR genes, DNMTs. MSI is the molecular fingerprint and a frequent phenomenon in cancers as the consequence of MMR genes mutations^50,51^. Emerging evidence revealed that most of the tumors with MSI-H/dMMR status exhibited high TMB^52,53^. These features are related to the increased neoantigen, affecting tumor-infiltrating lymphocytes and response to ICBs, thus could predict the response to immunotherapies independently^54–56^. TIGIT was reported to be positively correlated with MSI/dMMR in the colorectal cancer^57^. Our results not only showed the positive correlation of TIGIT with MSI/TMB in COAD, but also revealed more correlations between TIGIT expression and MSI/TMB in multiple other cancer types at pan-cancer level, such as in UCSC. However, the correlations of TIGIT expression with MSI and TMB didn’t coincide in some of the same cancer types, which could be explained by 2 reasons. First, though studies have shown the TMB elevation in tumors with MSI-H status, the correlation between MSI and TMB is still variable, which leads to the studies integrating the statuses of MSI and TMB for predicting the response to ICBs reported^58,59^. Studies focusing on the correlation between TIGIT and TMB in tumors are also lacking, which could be further investigated. Second, using different datasets and the peculiarities of each data collection method could lead to the different correlations of TIGIT with MSI and TMB in the same type of cancer. Besides genetic mutations, epigenetic alterations also impact the growth, proliferation, metastasis and immunosuppression of the tumors profoundly. DNA methylation is one of the most important epigenetic regulation. Aberrant levels of DNA methylation were associated with tumorigenesis and immune evasion in cancers^60^. Our results found certain positive and negative correlations between DNMTs and TIGIT expression in different cancer types, suggesting DNA methylation may also participate in the modulation of TIGIT, as previous studies reported^61,62^. Its mechanism is related to the reduced expression of the genes concerned with tumor suppression and anti-tumor immunity by DNA hypermethylation and overexpression of the genes responsible for tumorigenesis and immune suppression by DNA hypomethylation^63,64^. Altogether, different kinds of tumors and its immune microenvironment are driven by different methylation patterns, which is complicated and needs deeper investigation in the future. The relationship between DNMTs and TIGIT also indicates the possible strategy to target these checkpoint by methylation modulators or combine methylation modulators with ICBs to elevate the response rates^65,66^.

Considering all the results in different omics above, we could speculate that the genetic and epigenetic aberrant alterations initiate the tumorigenesis, which activates T cells and NK cells and gets infiltrated by immune cells. Then some T cells and NK cells upregulate TIGIT expression, which inhibits the immune function and leads to the immunosuppressive microenvironment in the tumors, promoting the development and metastases of the tumors and resulting in the poor prognosis.

As one of the most commonly targeted immune checkpoint^67^ and the core of a complex regulatory network included CD96, CD112R, CD226, CD155 and CD226^7^, TIGIT has been considered as a potential ICBs to develop novel immunotherapy strategies. Several preclinical studies have shown that TIGIT blockade alone could impede the growth and proliferation of the tumors^68–70^, even in anti-PD-1 resistant tumor model^8^. Moreover, combining the TIGIT blockade with PD-1 blockade^71,72^, IL-15 stimulation^73^ or optimized fractionated radiotherapy^74^, could promote the response to immunotherapy and increase the survival in animal models. Multiple clinical trials are also ongoing to test whether TIGIT blockade could translate into an actual benefit for patients with cancers (NCT04354246, NCT04150965, NCT04570839).

In this study, we showed the pan-cancer landscape of aberrant TIGIT expression across different tumors for the first time. Our findings will allow us to take the next step into a further functional investigation of TIGIT and clinical application of TIGIT blockade in specific cancers, providing new insights and options for the patients with cancers. Our study has several limitations. First, there’s no experimental validation of the predicted results. The relationship between the TIGIT expression and the nature of the tumors are needed to be validated in future experiments using the standardized methods. Second, more data from other public datasets are needed to validate our results.

## Supplementary Information

Supplementary Information 1.

Supplementary Information 2.

## Data Availability

The datasets for this study can be found in the TCGA Research Network (https://www.cancer.gov/tcga), GTEx (http://commonfund.nih.gov/GTEx/), and GEO (https://www.ncbi.nlm.nih.gov/geo/).
