# Supplementary figures and images for: A pan-cancer analysis revealing the role of TIGIT in tumor microenvironment
Source: Sci Rep. 2021 Nov 18;11:22502. doi: 10.1038/s41598-021-01933-9 (PMC8602416; doi:10.1038/s41598-021-01933-9)

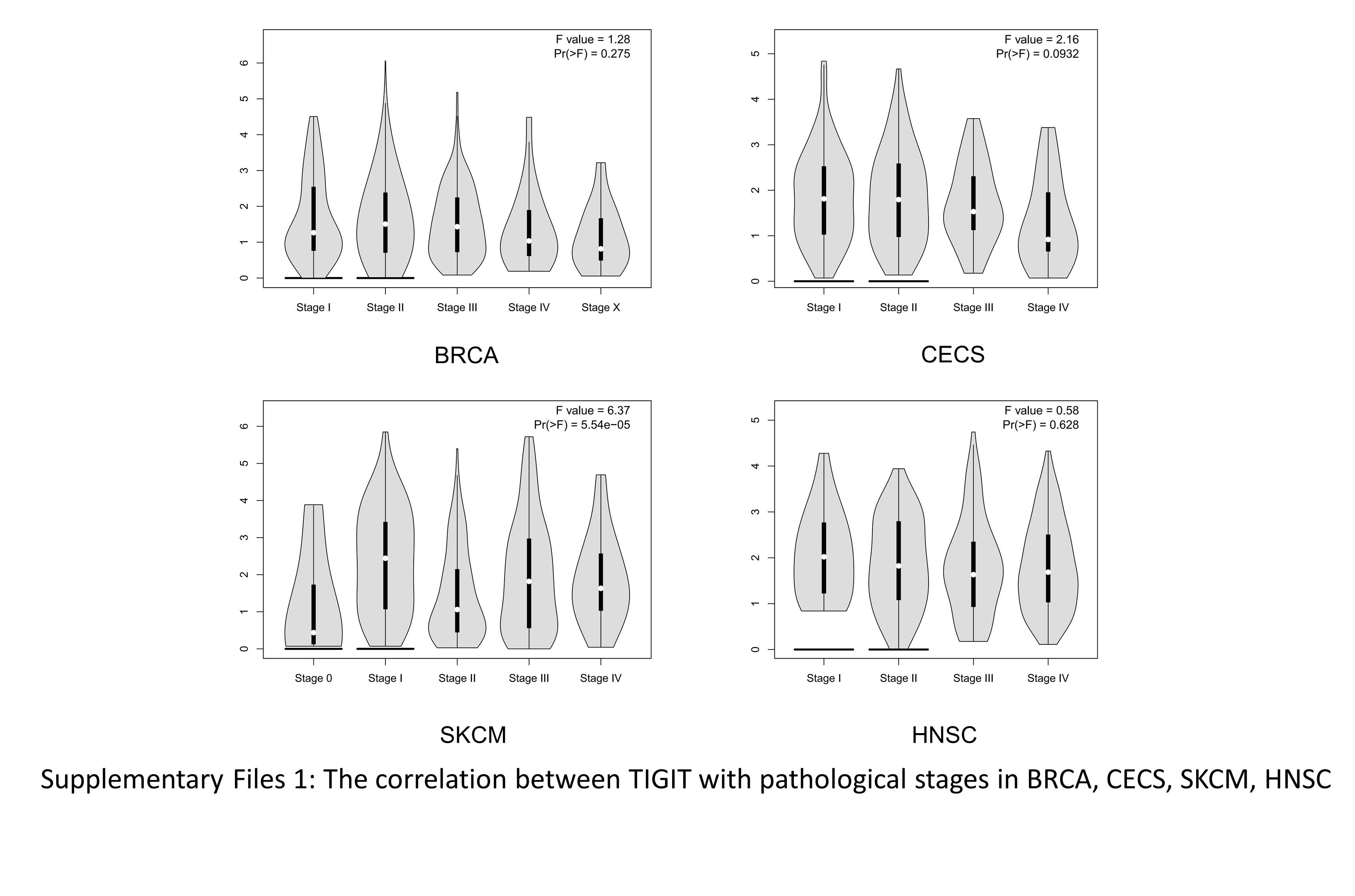

Supplement: Supplementary file 1 — Supplementary Information 1. [file 41598_2021_1933_MOESM1_ESM.jpg]

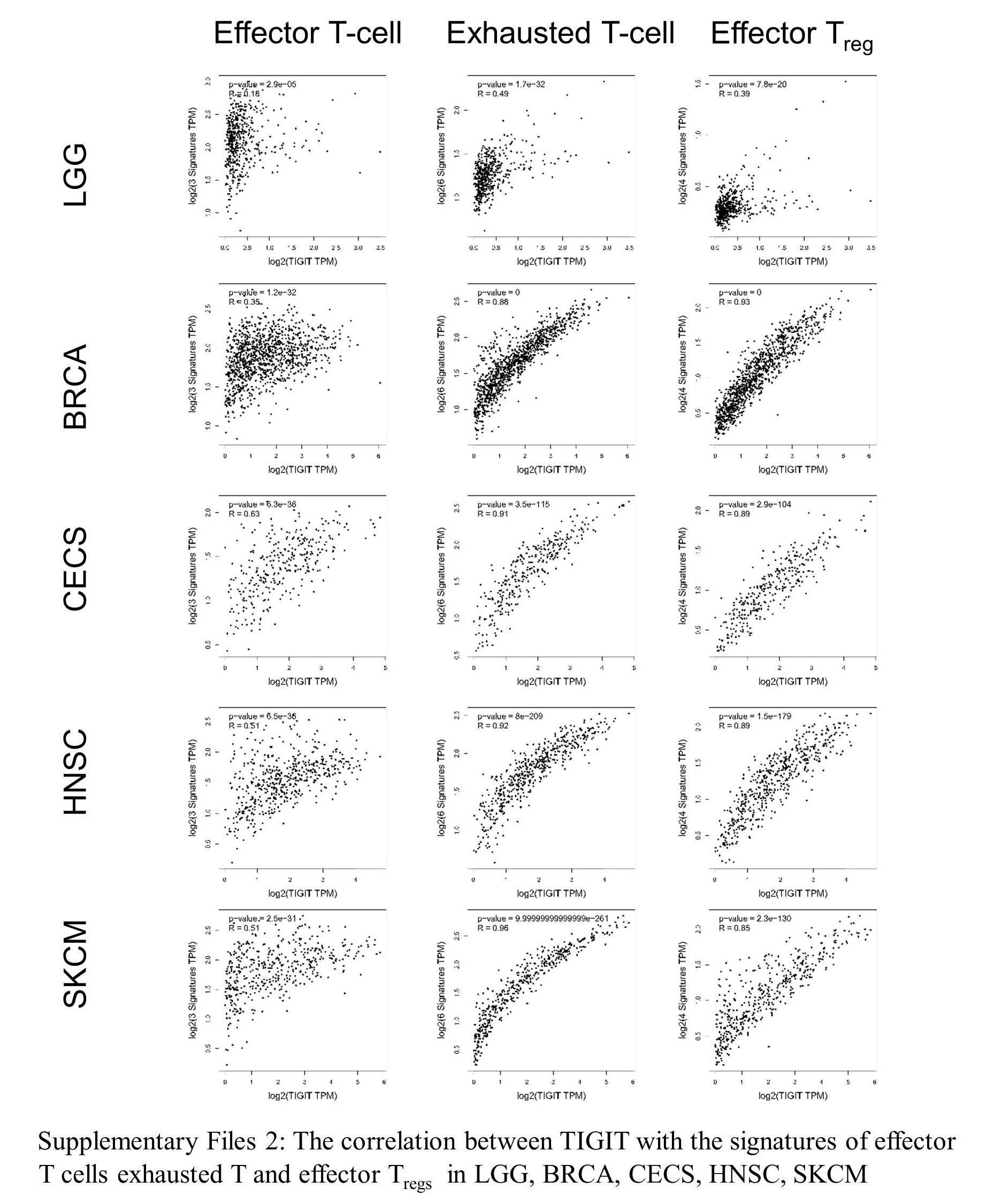

Supplement: Supplementary file 2 — Supplementary Information 2. [file 41598_2021_1933_MOESM2_ESM.jpg]
